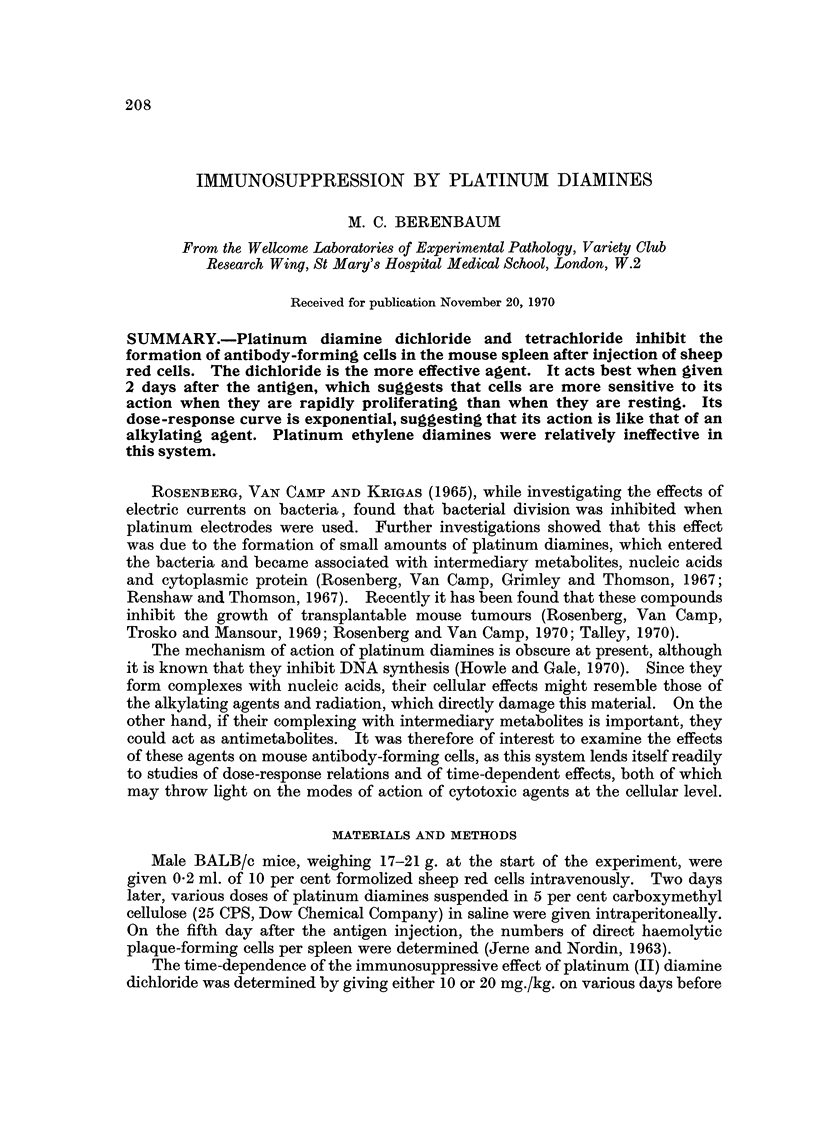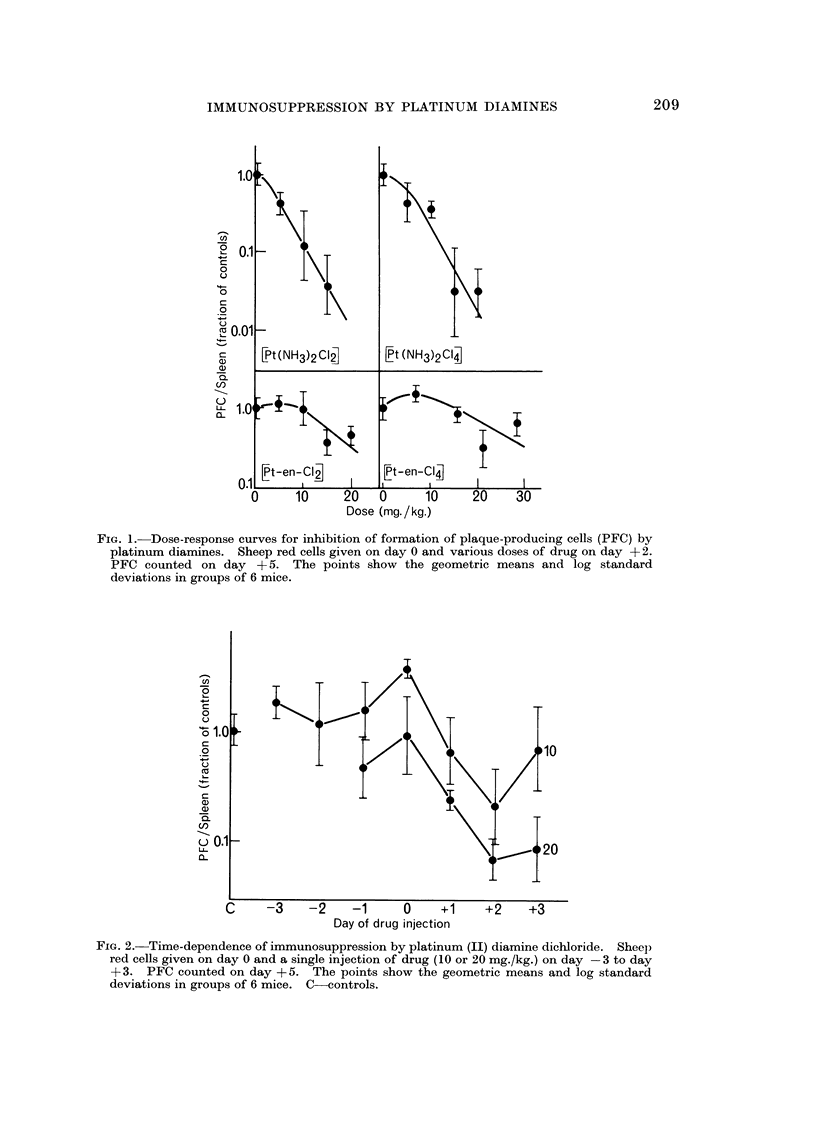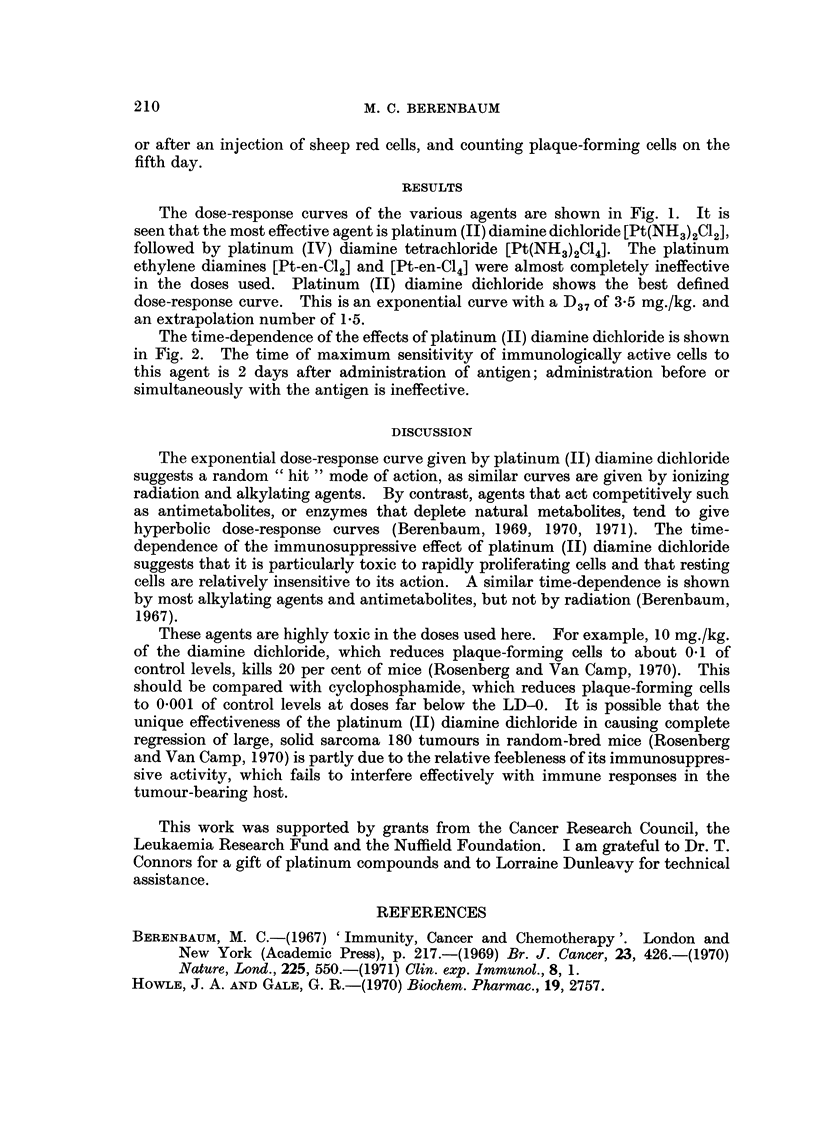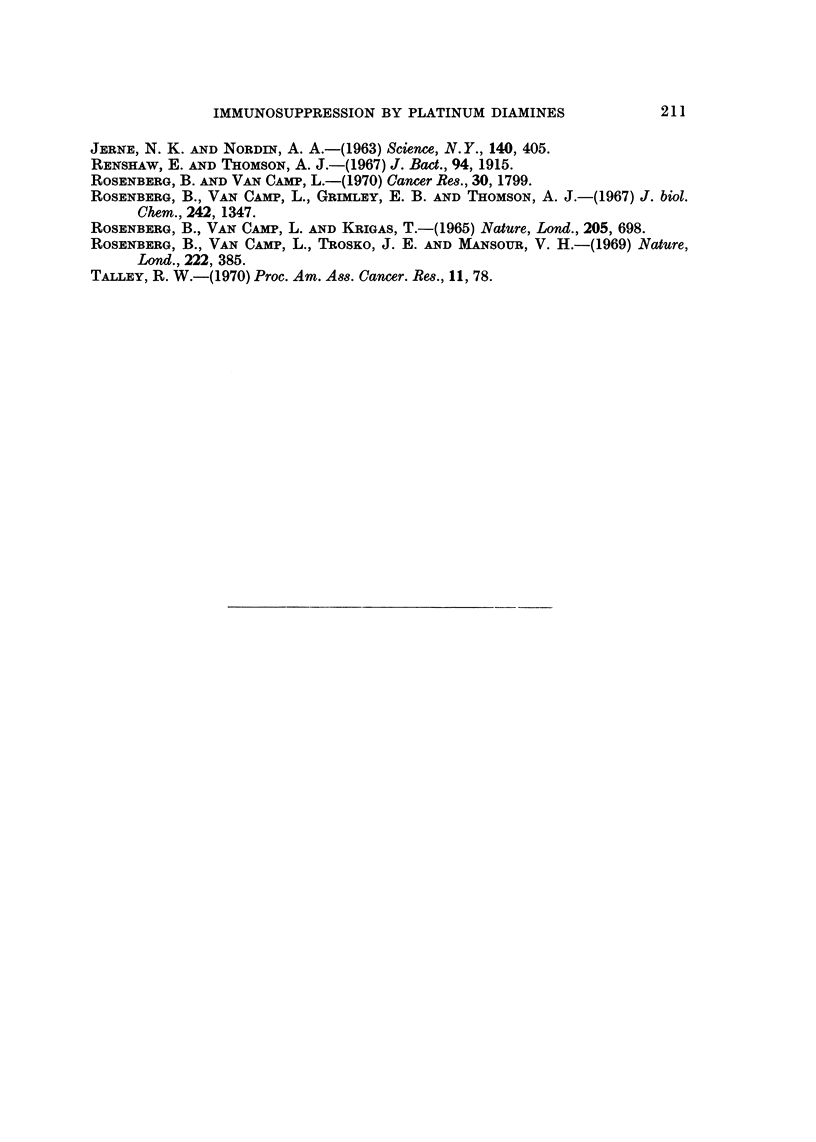# Immunosuppression by Platinum Diamines

**DOI:** 10.1038/bjc.1971.27

**Published:** 1971-03

**Authors:** M. C. Berenbaum

## Abstract

Platinum diamine dichloride and tetrachloride inhibit the formation of antibody-forming cells in the mouse spleen after injection of sheep red cells. The dichloride is the more effective agent. It acts best when given 2 days after the antigen, which suggests that cells are more sensitive to its action when they are rapidly proliferating than when they are resting. Its dose-response curve is exponential, suggesting that its action is like that of an alkylating agent. Platinum ethylene diamines were relatively ineffective in this system.


### IMMUNOSUPPRESSION BY PLATINUM DIAMINES

M. C. BERENBAUM

*From the Wellcome Laboratories of Experimental Pathology, Variety Club Research Wing, St Mary's Hospital Medical School, London, W.2*

Received for publication November 20, 1970

**SUMMARY.—Platinum diamine dichloride and tetrachloride inhibit the formation of antibody-forming cells in the mouse spleen after injection of sheep red cells. The dichloride is the more effective agent. It acts best when given 2 days after the antigen, which suggests that cells are more sensitive to its action when they are rapidly proliferating than when they are resting. Its dose-response curve is exponential, suggesting that its action is like that of an alkylating agent. Platinum ethylene diamines were relatively ineffective in this system.**

ROSENBERG, VAN CAMP AND KRIGAS (1965), while investigating the effects of electric currents on bacteria, found that bacterial division was inhibited when platinum electrodes were used. Further investigations showed that this effect was due to the formation of small amounts of platinum diamines, which entered the bacteria and became associated with intermediary metabolites, nucleic acids and cytoplasmic protein (Rosenberg, Van Camp, Grimley and Thomson, 1967; Renshaw and Thomson, 1967). Recently it has been found that these compounds inhibit the growth of transplantable mouse tumours (Rosenberg, Van Camp, Trosko and Mansour, 1969; Rosenberg and Van Camp, 1970; Talley, 1970).

The mechanism of action of platinum diamines is obscure at present, although it is known that they inhibit DNA synthesis (Howle and Gale, 1970). Since they form complexes with nucleic acids, their cellular effects might resemble those of the alkylating agents and radiation, which directly damage this material. On the other hand, if their complexing with intermediary metabolites is important, they could act as antimetabolites. It was therefore of interest to examine the effects of these agents on mouse antibody-forming cells, as this system lends itself readily to studies of dose-response relations and of time-dependent effects, both of which may throw light on the modes of action of cytotoxic agents at the cellular level.

### MATERIALS AND METHODS

Male BALB/c mice, weighing 17–21 g. at the start of the experiment, were given 0·2 ml. of 10 per cent formolized sheep red cells intravenously. Two days later, various doses of platinum diamines suspended in 5 per cent carboxymethyl cellulose (25 CPS, Dow Chemical Company) in saline were given intraperitoneally. On the fifth day after the antigen injection, the numbers of direct haemolytic plaque-forming cells per spleen were determined (Jerne and Nordin, 1963).

The time-dependence of the immunosuppressive effect of platinum (II) diamine dichloride was determined by giving either 10 or 20 mg./kg. on various days before

FIG. 1.—Dose-response curves for inhibition of formation of plaque-producing cells (PFC) by platinum diamines. Sheep red cells given on day 0 and various doses of drug on day +2. PFC counted on day +5. The points show the geometric means and log standard deviations in groups of 6 mice.

FIG. 2.—Time-dependence of immunosuppression by platinum (II) diamine dichloride. Sheep red cells given on day 0 and a single injection of drug (10 or 20 mg./kg.) on day −3 to day +3. PFC counted on day +5. The points show the geometric means and log standard deviations in groups of 6 mice. C—controls.

or after an injection of sheep red cells, and counting plaque-forming cells on the fifth day.

### RESULTS

The dose-response curves of the various agents are shown in Fig. 1. It is seen that the most effective agent is platinum (II) diamine dichloride [$Pt(NH_3)_2Cl_2$], followed by platinum (IV) diamine tetrachloride [$Pt(NH_3)_2Cl_4$]. The platinum ethylene diamines [Pt-en-$Cl_2$] and [Pt-en-$Cl_4$] were almost completely ineffective in the doses used. Platinum (II) diamine dichloride shows the best defined dose-response curve. This is an exponential curve with a $D_{37}$ of 3·5 mg./kg. and an extrapolation number of 1·5.

The time-dependence of the effects of platinum (II) diamine dichloride is shown in Fig. 2. The time of maximum sensitivity of immunologically active cells to this agent is 2 days after administration of antigen; administration before or simultaneously with the antigen is ineffective.

### DISCUSSION

The exponential dose-response curve given by platinum (II) diamine dichloride suggests a random " hit " mode of action, as similar curves are given by ionizing radiation and alkylating agents. By contrast, agents that act competitively such as antimetabolites, or enzymes that deplete natural metabolites, tend to give hyperbolic dose-response curves (Berenbaum, 1969, 1970, 1971). The time-dependence of the immunosuppressive effect of platinum (II) diamine dichloride suggests that it is particularly toxic to rapidly proliferating cells and that resting cells are relatively insensitive to its action. A similar time-dependence is shown by most alkylating agents and antimetabolites, but not by radiation (Berenbaum, 1967).

These agents are highly toxic in the doses used here. For example, 10 mg./kg. of the diamine dichloride, which reduces plaque-forming cells to about 0·1 of control levels, kills 20 per cent of mice (Rosenberg and Van Camp, 1970). This should be compared with cyclophosphamide, which reduces plaque-forming cells to 0·001 of control levels at doses far below the LD–0. It is possible that the unique effectiveness of the platinum (II) diamine dichloride in causing complete regression of large, solid sarcoma 180 tumours in random-bred mice (Rosenberg and Van Camp, 1970) is partly due to the relative feebleness of its immunosuppressive activity, which fails to interfere effectively with immune responses in the tumour-bearing host.

This work was supported by grants from the Cancer Research Council, the Leukaemia Research Fund and the Nuffield Foundation. I am grateful to Dr. T. Connors for a gift of platinum compounds and to Lorraine Dunleavy for technical assistance.

### REFERENCES

BERENBAUM, M. C.—(1967) 'Immunity, Cancer and Chemotherapy'. London and New York (Academic Press), p. 217.—(1969) *Br. J. Cancer*, **23**, 426.—(1970) *Nature, Lond.*, **225**, 550.—(1971) *Clin. exp. Immunol.*, **8**, 1.

HOWLE, J. A. AND GALE, G. R.—(1970) *Biochem. Pharmac.*, **19**, 2757.

JERNE, N. K. AND NORDIN, A. A.—(1963) *Science, N.Y.*, **140**, 405.

RENSHAW, E. AND THOMSON, A. J.—(1967) *J. Bact.*, **94**, 1915.

ROSENBERG, B. AND VAN CAMP, L.—(1970) *Cancer Res.*, **30**, 1799.

ROSENBERG, B., VAN CAMP, L., GRIMLEY, E. B. AND THOMSON, A. J.—(1967) *J. biol. Chem.*, **242**, 1347.

ROSENBERG, B., VAN CAMP, L. AND KRIGAS, T.—(1965) *Nature, Lond.*, **205**, 698.

ROSENBERG, B., VAN CAMP, L., TROSKO, J. E. AND MANSOUR, V. H.—(1969) *Nature, Lond.*, **222**, 385.

TALLEY, R. W.—(1970) *Proc. Am. Ass. Cancer. Res.*, **11**, 78.